# The Educational Program of Macrophages toward a Hyperprogressive Disease-Related Phenotype Is Orchestrated by Tumor-Derived Extracellular Vesicles

**DOI:** 10.3390/ijms232415802

**Published:** 2022-12-13

**Authors:** Serena Indino, Cristina Borzi, Claudia Moscheni, Patrizia Sartori, Loris De Cecco, Giancarla Bernardo, Valentino Le Noci, Francesca Arnaboldi, Tiziana Triulzi, Gabriella Sozzi, Elda Tagliabue, Lucia Sfondrini, Nicoletta Gagliano, Massimo Moro, Michele Sommariva

**Affiliations:** 1 Dipartimento di Scienze Biomediche per la Salute, Università degli Studi di Milano, Via Mangiagalli 31, 20133 Milan, Italy; 2 Tumor Genomics Unit, Department of Research, Fondazione IRCCS Istituto Nazionale dei Tumori, Via Venezian 1, 20133 Milan, Italy; 3 Dipartimento di Scienze Biomediche e Cliniche, Università degli Studi di Milano, Via G. B. Grassi, 74, L.I.T.A. Vialba, 20157 Milan, Italy; 4 Molecular Mechanisms Unit, Department of Research, Fondazione IRCCS Istituto Nazionale dei Tumori, Via Amadeo 42, 20133 Milan, Italy; 5 Molecular Targeting Unit, Department of Research, Fondazione IRCCS Istituto Nazionale dei Tumori, Via Amadeo 42, 20133 Milan, Italy

**Keywords:** hyperprogressive disease, macrophages, extracellular vesicles, anti-PD1 antibody, immune checkpoint inhibitors

## Abstract

Hyperprogressive disease (HPD), an aggressive acceleration of tumor growth, was observed in a group of cancer patients treated with anti-PD1/PDL1 antibodies. The presence of a peculiar macrophage subset in the tumor microenvironment is reported to be a sort of “immunological prerequisite” for HPD development. These macrophages possess a unique phenotype that it is not clear how they acquire. We hypothesized that certain malignant cells may promote the induction of an “HPD-related” phenotype in macrophages. Bone-marrow-derived macrophages were exposed to the conditioned medium of five non-small cell lung cancer cell lines. Macrophage phenotype was analyzed by microarray gene expression profile and real-time PCR. We found that human NSCLC cell lines, reported as undergoing HPD-like tumor growth in immunodeficient mice, polarized macrophages towards a peculiar pro-inflammatory phenotype sharing both M1 and M2 features. Lipid-based factors contained in cancer cell-conditioned medium induced the over-expression of several pro-inflammatory cytokines and the activation of innate immune receptor signaling pathways. We also determined that tumor-derived Extracellular Vesicles represent the main components involved in the observed macrophage re-education program. The present study might represent the starting point for the future development of diagnostic tools to identify potential hyperprogressors.

## 1. Introduction

Hyperprogressive disease (HPD) is a peculiar pattern of tumor progression characterizing a fraction of patients, affected by different types of cancers, that received immune checkpoint inhibitors (ICIs), such as anti-PD1/PDL1 antibodies [1,2]. Patients with HPD experience a strong acceleration of tumor growth and an increase in metastatic spread paralleled by a rapid deterioration of the clinical conditions. No consensus about the criteria to diagnose HPD has been reached yet and, therefore, it is not possible to precisely define the percentage of HPD patients among different studies. Moreover, although several works attempted to identify potential HPD-related biomarkers, mainly focusing on the possible association between the tumor mutational landscape and HPD occurrence, no diagnostic tools are available today [2]. 

There are several lines of evidence suggesting that HPD is a multifactorial phenomenon involving immune-related mechanisms but a full comprehension of the intricate cellular and molecular processes underlying this detrimental phenomenon is still missing. We previously reported that macrophages are the main immune cell population contributing to HPD development [3]. By analyzing non-small cell lung cancer (NSCLC) patient specimens, collected before the initiation of ICI therapy, we observed that the tumor lesions of HPD patients were infiltrated by particular clusters of tumor-associated macrophages (TAMs). Similar results were also obtained by performing in vivo experiments using immunodeficient mice. Mechanistically, we provided insights regarding the role of the Fc domain of the anti-PD1 antibody in promoting the functional reprogramming of macrophages, resulting in the acquisition of enhanced pro-tumor functions that, in turn, exacerbated cancer cell growth [3]. 

In the tumor microenvironment (TME), macrophages are exposed to a plethora of different stimuli exerting a strong influence on their biological function [4]. In this context, the ability of cancer cells to “educate” macrophages is well-recognized, through the release of immunomodulatory factors, such as cytokines or metabolites, making these innate immune cells potential allies in sustaining and supporting tumor growth [5]. Since our previous findings suggested that the presence of a specific population of TAMs before ICI administration appears to be a prerequisite for HPD onset [3], we hypothesized that certain tumor cells may induce the acquisition of an “HPD-associated” phenotype that can unleash HPD upon interaction with anti-PD1 antibody in macrophages. In the present study, we found that human NSCLC cell lines reported undergoing an HPD-like development following anti-PD1 antibody administration in preclinical models [3], polarized macrophages toward a phenotype that is completely different from that induced by other NSCLC cell lines. This phenotype is highly pro-inflammatory but is characterized by both M1 and M2 features. Finally, we identified Extracellular Vesicles (EVs), in the range size of exosomes, as players involved in the observed macrophage re-education program.

## 2. Results

### 2.1. Conditioned Medium (CM) from NSCLC Cell Lines Undergoing an HPD-like Tumor Growth in Mice after PD1 Blockade Polarizes Macrophages towards a Peculiar Phenotype

#### 2.1.1. Effect of NSCLC CMs on Macrophage Gene Expression Profiles

Considering that only a fraction of NSCLC patients experience HPD under PD1 blockade therapy [2] and that macrophages play a pivotal role in HPD development [3], we postulated the existence of an “HPD-related” macrophage phenotype, possibly shaped by tumor cells through the release of soluble factors. To address this question, bone marrow-derived macrophages (BMDMs) were exposed for 24 h to the CMs obtained from five different NSCLC cell lines, representative of the most frequent genetic alterations in NSCLC [6] (Table S1), and a comprehensive gene expression profile was performed. H460 and PC9 cells were chosen because they showed an increase in tumor growth after anti-PD1 antibody treatment in immunodeficient mice [3], while H1299 and A549 cell lines did not (unpublished data and [7,8], respectively). A549 cells are similar to the H460 cell line, at least when considering the mutational landscape. Calu-1 cells have a mutation in the *KRAS* gene, as H460 and A549 cells, and a wild-type *LKB1* gene and a *TP53* deletion, as the H1299 cell line [9,10,11,12]. 

**Figure 1:**
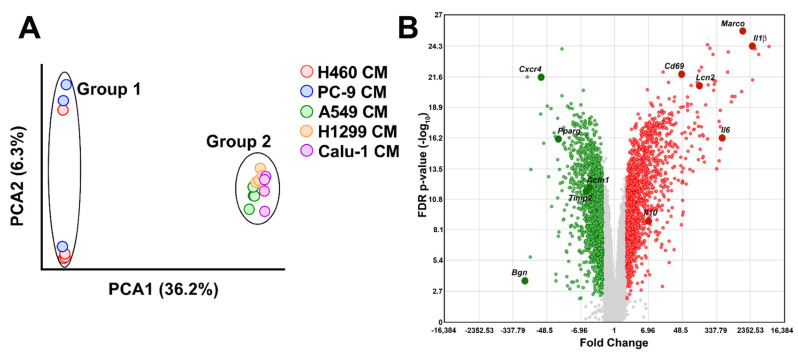
Impact of NSCLC cell line CMs on BMDMs gene expression profile. (**A**) Global representation of normalized gene expression profile data of BMDMs exposed to the different CMs by PCA. Experimental groups segregated into two clusters defined as Group 1 (H460- and PC9-CMs stimulated macrophages) and Group 2 (A549-, H1299- and Calu-1-CMs stimulated macrophages). Each dot represents a sample. (**B**) Volcano plot of transcripts with FDR < 0.01 and −2 ≤ FC ≥ 2 resulted from the comparison between Group 1 and Group 2 BMDM gene profile data. In total, 1420 and 1626 transcripts are up- (red dots) and down-regulated (green dots), respectively. Plot shows the Fold Change on the *X*-axis versus FDR *p*-value (on a log10 scale) on the *Y*-axis. NSCLC: Non-small cell lung cancer; BMDMs: bone marrow-derived macrophages; PCA: principal component analysis.

To detect similarities and dissimilarities among the experimental groups, a principal component analysis (PCA) was performed on the global gene expression profile data. CM-stimulated macrophages were segregated into two distinct clusters, named Group 1 and Group 2. The former comprises BMDMs incubated with H460- and PC9-CMs, the latter comprises all the other samples (Figure 1A). Among the 65,957 probes present in the Clariom™ D Array Platform, a total of 3046 transcripts resulted statistically differentially regulated with a False Discovery Rate (FDR) < 0.01 and a −2 ≤ Fold-change (FC) ≥ 2 in Group 1 compared to Group 2 macrophages. In total, 1420 transcripts were found to be up-regulated while 1626 were down-regulated. The complete list is provided in Table S2. The volcano plot shows the results of the differential gene expression analysis (Figure 1B). 

**Figure 2:**
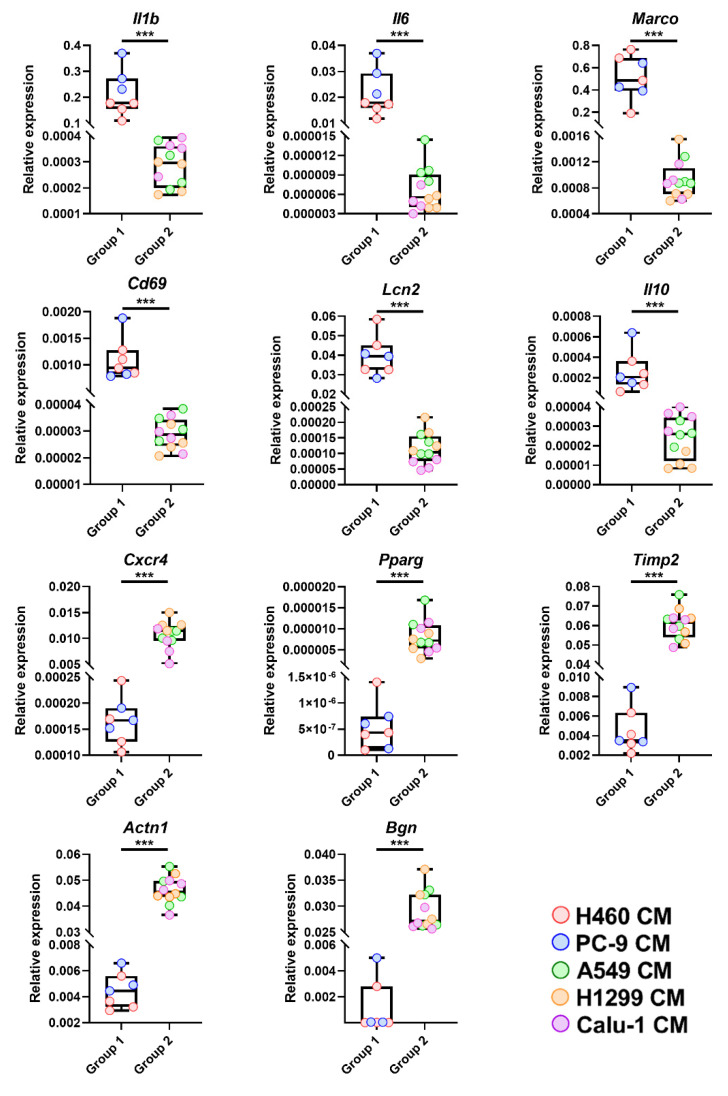
Validation of gene expression profile data by real-time PCR. mRNA level of *Il1β*, *Il6*, *Marco*, *Cd69*, *Lcn2*, *Il10*, *Cxcr4*, *Pparg*, *Timp2*, *Actin1* and *Bgn*, selected from the first quartile of the most up- (*Il1β*, *Il6*, *Marco*, *Cd69*, *Lcn2*, *Il10*) or down- (*Cxcr4*, *Pparg*, *Timp2*, *Actin1*, *Bgn*) regulated genes between Group 1 and Group 2 BMDMs (FDR < 0.01), was analyzed by real-time PCR. Results are presented as 2^−ΔCt^. *** *p* < 0.001 by two-tailed unpaired Student’s *t*-test or the Mann–Whitney U test for nonparametric distribution of data.

For gene profiling validation, mRNA expression levels of Interleukin 1β (*Il1β*), Interleukin 6 (*Il6*), Macrophage receptor with collagenous structure (*Marco*), Cluster of Differentiation 69 (*Cd69*), Lipocalin 2 (*Lcn2*), Interleukin 10 (*Il10*), Chemokine (C-X-C motif) receptor 4 (*Cxcr4*), Peroxisome proliferator-activated receptor gamma (*Pparg*), Tissue inhibitor of metalloproteinase 2 (*Timp2*), Actinin alpha 1 (*Actn1*) and Biglycan (*Bgn*) (Figure 1B) were quantified by real-time PCR. These molecules, all involved in regulating macrophage activation and function [13,14,15,16,17,18,19,20,21,22], were selected among the first quartile of genes with the highest or lowest FC resulting from the comparison between Group 1 and Group 2 BMDMs (FDR < 0.01, Figure 1B). Real-time PCR results validated the microarray data (Figure 2).

#### 2.1.2. Pathways Induced in Macrophages after Exposure to the Different CMs

**Figure 3:**
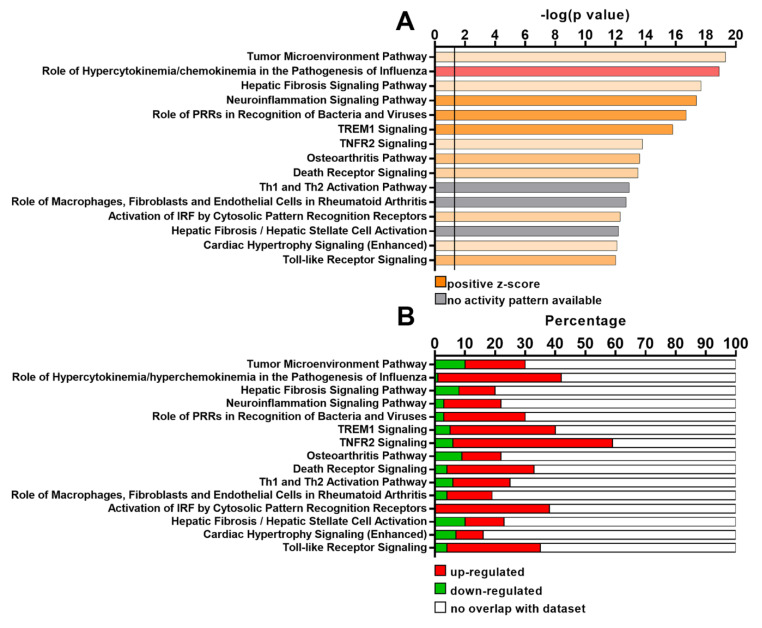
Functional analysis of BMDMs exposed to NSCLC cell line CMs by IPA. (**A**) Top 15 differentially regulated canonical pathways between Group 1 and Group 2 BMDMs resulted from IPA “Core Analysis” performed on annotated transcripts with FDR < 0.01 and −2 ≤ FC ≥ 2. The identified pathways are represented on the *y*-axis. On the *x*-axis, -log(*p*-value) as determined by Fisher’s exact test, is shown. The black straight line indicates the minimum significance level. (**B**) Stacked bar chart showing the percentage of up-regulated (red), down-regulated (green) and not overlapping (white) genes retrieved from our microarray analysis in each canonical pathway.

Ingenuity Pathway Analysis (IPA) revealed that pathways related to cytokine release (i.e., the role of hypercytokinemia/chemokinemia in the pathogenesis of influenza), inflammation (i.e., neuroinflammation signaling pathway) and Toll-like receptors (TLR) signaling (i.e., the role of PPRs in recognition of bacteria and viruses, activation of interferon regulatory factor–IRF-by cytosolic Pattern Recognition receptors, Toll-like receptor signaling) were all over-represented in Group 1 BMDMs (Figure 3A,B). In the same group, we could also observe the up-modulation of the TREM1 signaling pathway (Figure 3A,B). 

**Figure 4:**
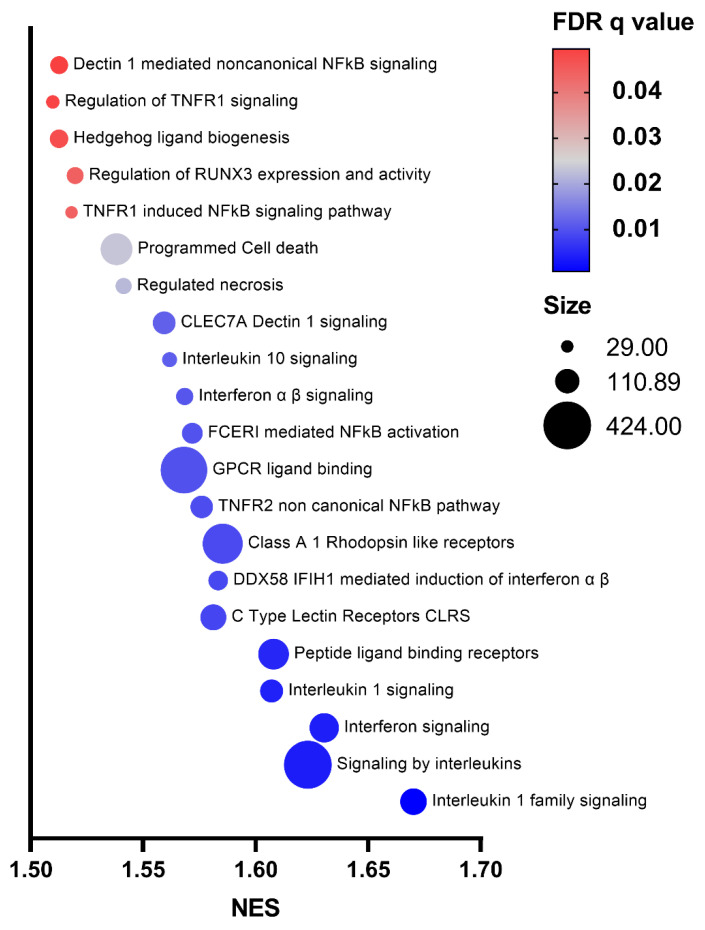
List of pathways emerged from GSEA analysis of Group 1 and Group 2 BMDMs. Bubble plot of the top 21 significant Reactome pathways (FDR *q*-value < 0.05) enriched in Group 1 macrophages as determined by GSEA analysis. The *X*-axis represents the normalized enrichment score (NES) and *Y*-axis indicates enriched pathway terms. Bubble area is proportional to the size of the gene set. Bubble color indicates the FDR *q*-value.

To strengthen these findings, Gene Set Enrichment Analysis (GSEA), a type of functional analysis that considers the global transcriptomic profile and is not restricted to DEGs [23], was applied to the gene profile data. The results obtained by GSEA showed enrichment of 21 gene sets with an FDR *q*-value < 0.05 in Group 1 compared to Group 2 macrophages (Figure 4). In line with previous findings, the majority of these pathways were related to innate immune recognition receptors or to inflammatory processes, such as interleukin-1 or interferon signaling. However, we could also observe a gene set associated with *Il10* signaling, possibly indicating the co-existence of M1 and M2 features (Figure 4).

#### 2.1.3. Analysis of Macrophage Phenotype after Exposure to the Different CMs

**Figure 5:**
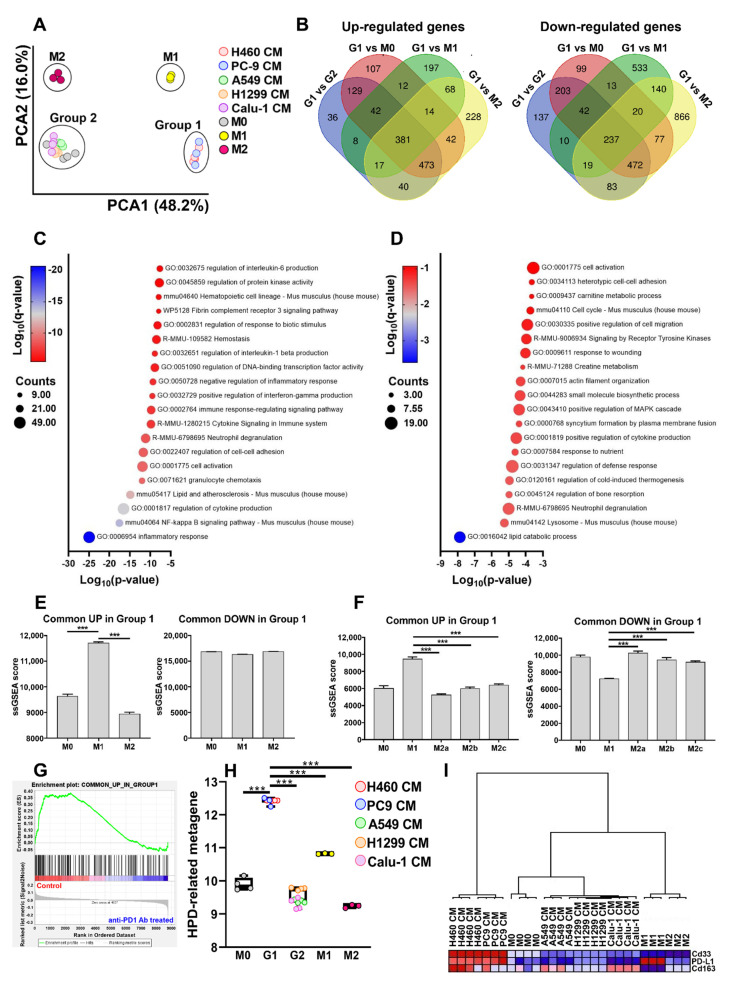
Analysis of NSCLC cell-mediated polarization skewing of BMDMs by bioinformatic analysis. (**A**) Global representation of normalized gene expression profile data of M0 (unstimulated), M1 (LPS + IFN-γ), M2 (IL-4) and NSCLC cell CM-stimulated macrophages by PCA. Each dot represents a sample. (**B**) Venn diagram showing the overlap between up- or down-modulated DEGs (FDR < 0.01 and −2 ≤ FC ≥ 2) emerged by comparing Group 1 to M0, M1, M2 and Group 2 macrophages. G1: Group 1 macrophages; G2: Group 2 macrophages. (**C**) Bubble plot of the top 20 clusters with their representative enriched terms (one per cluster) emerged by Metascape analysis of the “Common UP in Group 1” gene list. The *X*-axis represents the *p*-value in log base 10 and *Y*-axis indicates enriched pathway terms. Bubble size is proportional to the count that is the number of genes in the user-provided lists with membership in the given ontology term. Bubble color represents Log10(*q*-value) which is the multi-test adjusted *p*-value in log base 10. (**D**) Bubble plot of the top 20 clusters with their representative enriched terms (one per cluster) emerged by Metascape analysis of the “Common DOWN in Group 1” gene list. The *X*-axis represents the *p*-value in log base 10 and *Y*-axis indicates enriched pathway terms. Bubble size is proportional to the count that is the number of genes in the user-provided lists with membership in the given ontology term. Bubble color represents Log10(*q*-value) which is the multi-test adjusted *p*-value in log base 10. (**E**) ssGSEA enrichment scores of “Common UP in Group 1” and “Common DOWN in Group 1” gene sets in M0 (unstimulated), M1 (LPS + IFN-γ) and M2 (IL-4) macrophages. (**F**) ssGSEA enrichment scores of “Common UP in Group 1” and “Common DOWN in Group 1” gene sets in different macrophage subtypes. Gene expression data were retrieved from GSE32690. (**G**) GSEA enrichment plot of “Common UP in Group 1” gene set applied to gene expression profile data of H460 xenografts treated with saline (Control) or with anti-PD1 antibody (anti-PD1 treated). (**H**) Expression of “HPD-related metagene” in Group 1, Group 2, M0, M1 and M2 polarized BMDMs. G1: Group 1 macrophages; G2: Group 2 macrophages. (**I**) Hierarchical clustering based on the expression level of *Cd33*, *PD-L1* and *Cd163* genes retrieved from normalized gene expression profile data of Group 1, Group 2, M0, M1 and M2 polarized BMDMs. Data are presented as the mean ± SEM. *** *p* < 0.001 by One-way ANOVA followed by Tukey’s multiple comparison test.

The phenotype acquired by macrophages exposed to the different NSCLC cell line CMs was initially evaluated by PCA. Gene profiling data of unstimulated (M0) and LPS + IFN-γ− (M1) or IL-4- (M2) polarized BMDMs were also included in the analysis. Although Group 1 and Group 2 were segregated into two independent clusters, it is possible to observe that Group 1 and Group 2 BMDMs were located in proximity to M1 and M2 macrophages, respectively. M0 BMDMs were found comprised in Group 2 macrophages (Figure 5A). DEGs, which emerged by comparing Group 1 macrophage gene profile data to every other experimental group (Table S3), were intersected in order to identify genes always up- or down-regulated in Group 1 in comparison to Group 2, M0, M1 and M2 macrophages. As shown in Figure 5B, 381 and 237 unique annotated transcripts resulted in being commonly up- and down-modulated in Group 1 BMDMs, respectively. These gene lists served to generate two gene sets, named “Common UP in Group 1” and “Common DOWN in Group 1”, utilized in subsequent bioinformatic functional analyses (Table S4). Metascape analysis revealed that pathways mostly related to inflammatory response, immune cell activation and cytokine production were represented in the “Common UP in Group 1” gene list (Figure 5C) while in the “Common DOWN in Group 1” we could observe the prevalence of biological processes involved in metabolism and cytoskeleton reorganization (Figure 5D). In the “Common UP in Group 1” list, we found genes encoding for several cytokines (*Il12a*, *Il12b*, *Il18*, *Il1α*, *Il1β*, *Il6*, *Tnf*, *Il10*), chemokines (*Ccl3*, *Ccl4*, *Cxcl1*, *Cxcl2*, *Cxcl3*, *Cxcl5*) and the pro-angiogenic factor *Vegfa* (Table S4 and Figure S1A). Interestingly, *Il6* and *Il10* mRNA levels, selected as representatives of pro- and anti-inflammatory mediators, were sufficient to separate Group 1 macrophages from all the other groups (Figure S1B). These results may indicate that soluble factors released by H460 and PC9 cells are able to induce an M1-like pro-inflammatory phenotype in Group 1 BMDMs that simultaneously express M2-related markers. Subsequently, these two gene sets were exploited to quantify their enrichment levels in the canonical M0, M1 and M2 macrophage phenotypes. A statistically significant enrichment of the gene set “Common UP in Group 1” was observed in M1 macrophages compared to M0 and M2 (Figure 5E). No differences were found in the three macrophage subsets when the analysis was performed using the “Common DOWN in Group 1” gene set (Figure 5E). We applied the same analysis to a publicly available dataset (GSE32690) that comprises several types of polarized macrophages [24]. In line with previous data, the single sample GSEA (ssGSEA) score showed a higher value in M1 macrophages compared to all the other subsets when the “Common UP in Group 1” gene set was applied to the dataset. Accordingly, the genes constituting the “Common DOWN in Group 1” gene set were less represented in M1 macrophages compared to the other subpopulations (Figure 5F).

Moreover, we performed GSEA analysis on the microarray data obtained from H460 xenografts treated (or not) with anti–PD1 antibody, as described in [3]. Since a statistically significant enrichment (FDR *q*-value < 0.05) of the “Common UP in Group 1” gene set was found in untreated tumors, these results may indicate that H460 cells possess the ability to shape macrophage polarization not only in vitro but also in vivo and possibly suggests that anti-PD1 antibody treatment can further modify the macrophage gene expression profile (Figure 5G). 

We previously reported that a peculiar population of tumor-infiltrating macrophages, characterized by the co-expression of CD163, CD33 and PD-L1, was present in the tumor microenvironment of HPD patients [3]. These three markers were utilized to create an “HPD-related metagene”, whose expression was then evaluated in Group 1, Group 2, M0, M1 and M2 BMDMs. Group 1 macrophages showed the highest level of the metagene score even compared to M1 macrophages (Figure 5H). Moreover, these three genes alone were able to segregate Group 1 macrophages from all the other groups, as determined by hierarchical clustering (Figure 5I). Collectively, these findings suggest that soluble factors released by H460 and PC9 tumor cells are able to promote a switch towards a polarization profile resembling the pro-inflammatory M1 phenotype in macrophages but that, however, maintains its peculiar distinctiveness.

### 2.2. Extracellular Vesicles (EVs) Are Responsible for Macrophage Reprogramming 

**Figure 6:**
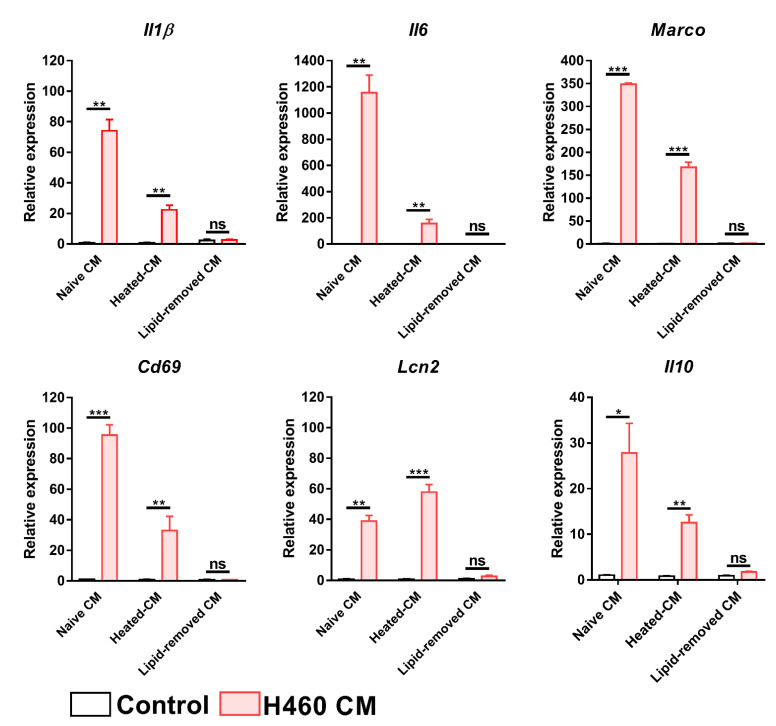
Effect of protein and lipid removal from H460 CM on BMDMs. mRNA level of *Il1β*, *Il6*, *Marco*, *Cd69*, *Lcn2* and *Il10* genes, as determined by Real-Time PCR, after BMDMs exposure to H460 heated-CMs or lipid removed-CMs. Data were normalized to the housekeeping gene expression level (*β2m*) and analyzed by the comparative 2^−ΔΔCt^ method. Data are presented as the mean ± SEM. * *p* < 0.05, ** *p* < 0.01, *** *p* < 0.001 by unpaired *t*-test; ns: not significant.

To investigate which components of the CM may mediate the effect on macrophage transcriptome, BMDMs were incubated for 24 h with an H460 CM in which the protein or the lipid/cholesterol contents were removed. At the end of treatment, the mRNA level of *Il1β*, *Il6*, *Marco*, *Cd69*, *Lcn2* and *Il10* was assessed by real-time PCR. Although heated CM induced the up-modulation of almost all the considered genes, its impact on macrophages was less potent compared to that of untouched CM (Figure 6). On the contrary, *Lcn2* expression appeared to be higher in heated CM compared to naïve CM-treated macrophages. In particular circumstances of cell stress, *Lcn2* can be transcriptionally activated by *Nupr1 [25]*, a gene up-regulated in Group 1 versus Group 2 BMDMs (Table S2). Real-time PCR analysis revealed that the mRNA of *Nupr1* and its downstream target *Trib3 [26]* is equally induced by naïve and heated CM (Figure S2). Notably, a complete abrogation of mRNA induction was observed in the absence of the lipid/cholesterol content (Figure 6). These results indicate that the molecule(s) involved in this process may possess a lipid-based structure. 

**Figure 7:**
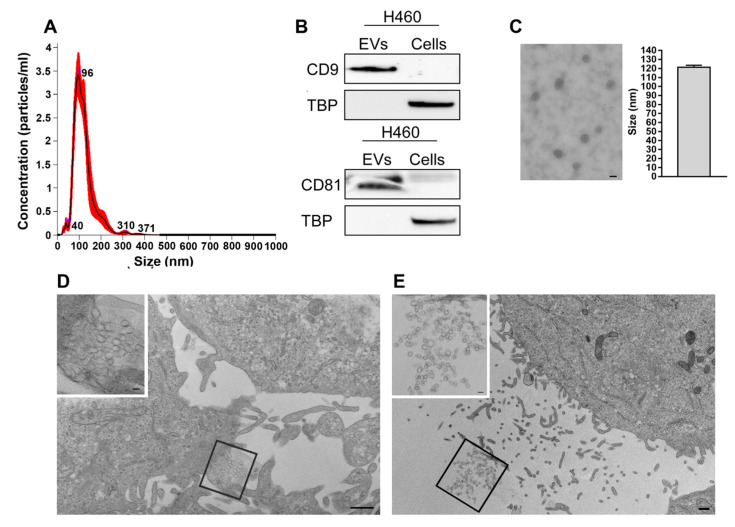
Characterization of EVs isolated from H460 cell line. (**A**) Size distribution of H460-derived EVs determined by NTA (NanoSight NS300 instrument-Malvern Panalytical). (**B**) Immunoblotting of the exosome-enriched proteins CD9 and CD81. H460 cellular lysates were used as a control for western blot analysis. TBP: TATA-binding protein, nuclear marker. (**C**) Representative electron microscopy image of EVs isolated from the conditioned media of H460 cells. EVs show the typical cup-shaped or round morphology of exosomes, as well as a compatible average diameter (≈120 nm). Scale bar = 100 nm. (**D**) Representative TEM image showing a new cluster of EVs enclosed by a membrane expelled by an H460 cell. Scale bar = 500 nm. In the upper left an enlargement of the boxed area. Scale bar = 100 nm. (**E**) Representative TEM image exhibiting scattered EVs in the extracellular milieu between H460 cells. Scale bar = 500 nm. In the upper left a greater enlargement of the outlined area. Scale bar = 100 nm.

Since Extracellular Vesicles (EVs) were also cleared from the CM by the lipid-removal agent (see Material and Methods section) and EVs released by tumor cells can have a role in macrophage polarization [27], EVs from H460 CM (H460-EVs) were isolated by ultracentrifugation and then incubated with BMDMs. EVs’ characterization by Nanoparticle Tracking analysis (NTA) revealed an EV enrichment within the size range of exosomes (<160 nm) [28] (Figure 7A), (average mean size: 128 ± 7 nm; average mode size: 98 ± 4 nm; average % of EVs < 160 nm: 80 ± 3, Table S5), Accordingly, EV-enriched tetraspanins, CD9 and CD81, were clearly detected in the EV pellet. The purity of the isolates was confirmed by the absence of the nuclear marker protein TATA Binding protein (TBP) (Figure 7B). Moreover, H460 EVs were also analyzed by transmission electron microscopy (TEM). Vesicles showed a cup-shaped or round morphology compatible with exosomes and an average diameter size of ≈120 nm (Figure 7C), confirming the NTA results. Ultra-thin sections of 2D-monolayer cultures in situ on a Petri dish of the H460 cell line were also investigated. In the extracellular milieu, we observed clusters of endomembrane vesicles enclosed by a membrane budding from the plasma membrane (Figure 7D), as well as free EVs (Figure 6E), likely released following the fusion of the intracellular multivesicular endosomes with the plasma membrane. The mean diameter of all the vesicles found in the extracellular space was compatible with that of the EVs isolated from the conditioned media (data not shown).

**Figure 8:**
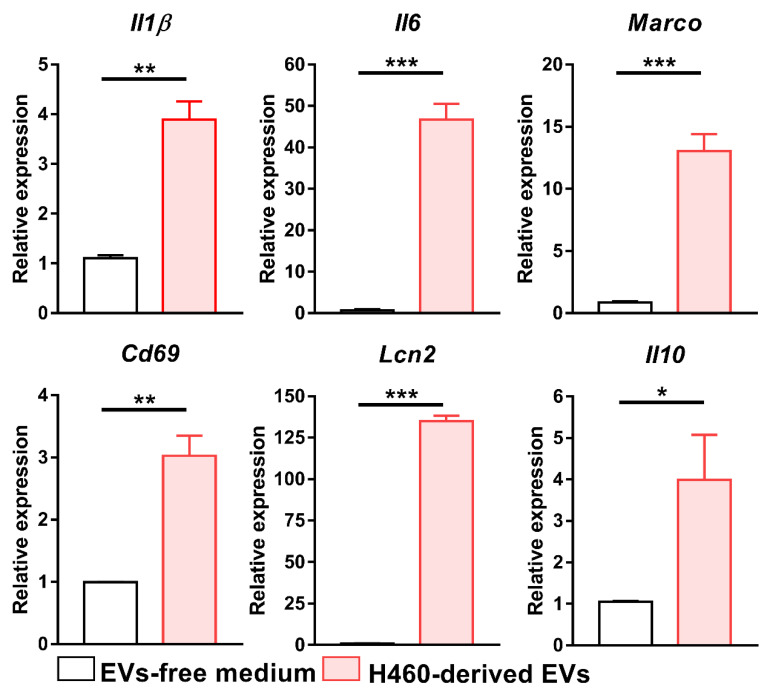
Effect of H460 EVs on BMDMs. mRNA level of *Il1β*, *Il6*, *Marco*, *Cd69*, *Lcn2* and *Il10* genes, as determined by Real-Time PCR after BMDM exposure to H460-derived EVs, isolated by ultracentrifugation. Data were normalized to the housekeeping gene expression level (*β2m*) and analyzed by the comparative 2^−ΔΔCt^ method. Data are presented as the mean ± SEM. * *p* < 0.05, ** *p* < 0.01, *** *p* < 0.001 by unpaired *t*-test.

Finally, we evaluated the impact of H460 EVs on macrophages. As shown in Figure 8, the exposure of BMDMs to H460-derived EVs was able to determine the up-modulation of all the considered genes compared to macrophages treated with an EV-free medium. Moreover, the incubation of RAW264.7 mouse macrophage cells with H460 and PC9 EVs resulted in statistically significant increased levels of *Il1β*, *Il6*, *Cd69* and *Lçn2* as compared to those treated with H1299 EVs (Figure S3). A similar experiment conducted with THP-1-derived human macrophages showed the up-regulation of *IL1β*, *IL-6* and *MARCO* (Figure S4). These results suggest that EVs play a pivotal role in shaping macrophages toward a phenotype related to HPD occurrence

## 3. Discussion

### 3.1. Macrophages Characterized by an “HPD-Related” Phenotype Are Pro-Inflammatory

HPD is a complex phenomenon, characterizing a percentage of cancer patients treated with ICIs, that rapidly leads to death. Although many studies have attempted to unravel its cellular and molecular mechanisms, there is still uncertainty surrounding the origins of HPD and whether they are already present in the TME before the initiation of the therapy [2]. The definitive answer to this biological and clinical question is extremely important for a better comprehension of this detrimental pattern of progression and for the discovery of potential biomarkers for prevention. In the present work, we provide insights that EVs released by tumor cells reprogram macrophages towards a phenotype that, in vivo, is able to trigger HPD upon interaction with an anti-PD1 antibody. Since we previously reported that macrophages are involved in HPD development [3], in this work, we explored the possibility that tumor cells can elicit an “HPD-prone” phenotype in macrophages through the release of soluble factors. Exploiting the CMs of five different NSCLC cell lines, we were able to observe that macrophages stimulated with the CMs of H460 and PC9 cells, reported to undergo an HPD-like tumor growth after anti-PD1 antibody administration in vivo [3], showed a gene expression profile completely different from that of BMDMs incubated with the CMs obtained from the other cell lines. The differences in terms of macrophage stimulatory capabilities seem to be independent of the tumor mutational landscape. Indeed, H460 and A549 cells, although possessing similar mutations [10], have an opposite effect on macrophage phenotype. These findings are in line not only with our previous work [3] but also with a recent study showing that the effect of NSCLC cell lines on macrophage phenotype appears not to correlate with the tumor cell genotype or with other clinicopathological characteristics [29]. However, we cannot exclude that still unidentified genomic alterations(s) could be associated with the effects observed on macrophages. It is also plausible to speculate that other tumor intrinsic factors may dictate the ability of cancer cells to induce HPD even through macrophage reprogramming. For instance, it was recently demonstrated that, in tumor cells, NLRP3 signaling, a key player of the inflammasome pathway [30], can trigger HPD after anti-PD1 antibody administration [31]. We next investigated the phenotype acquired by macrophages after exposure to the different NSCLC cell line CMs. PCA and ssGSEA analyses suggested that Group 1 BMDMs possess a phenotype that may resemble M1 macrophages. Since M1 macrophages are usually associated with anti-tumor immune response [32], our findings may appear counterintuitive. Indeed, it may be difficult to understand the reason why cancer cells may “educate” the immune system towards a phenotype potentially harmful to the tumor itself. However, we should carefully consider and interpret which kind of pro-inflammatory profile we are facing. It is well known that for some neoplastic diseases, including lung cancer, a strong inflammatory environment can sustain tumor initiation and development [33]. For instance, Group 1 BMDMs are characterized by the over-expression of several pro-inflammatory cytokines, such as *Il1β*, *Il6*, *Tnf*. IL1β is a cytokine able to induce angiogenesis, tumor growth and metastasis [34,35]. The evident pro-tumor role of this molecule is testified by several clinical trials investigating IL1β as a promising therapeutic target for the treatment of lung cancer patients [34,35]. On the same line, IL6 can promote tumor cell expansion and, accordingly, elevated levels of this cytokine were reported to correlate with an increased risk of cancer progression in NSCLC patients [36,37]. TNF, although initially discovered to induce cancer cell death, can also possess pro-tumor properties [38]. Moreover, these three cytokines secreted by macrophages can synergistically favor cancer stem cell survival and renewal and maintain stem cell niche [39]. In some circumstances, they can also mediate immunosuppression, creating a more favorable environment for tumor progression [40,41,42]. Therefore, these pro-inflammatory cytokines can act on two fronts by directly promoting cancer cell proliferation and by dampening the immune response against tumors. 

The notion that Group 1 BMDMs are characterized by a pro-inflammatory profile is also corroborated by GSEA analysis. Indeed, the Trem1 signaling pathway was found enriched in Group 1 macrophages. Trem1 is a receptor expressed by innate immune cells, including macrophages, and it is reported to amplify the inflammatory response in infectious and non-infectious diseases, such as cancer [43]. Trem1 on myeloid cells is associated with tumor progression and poor prognosis [44,45], and the blockade of this receptor on TAMs is able to ameliorate immunosuppression and revert ICI resistance [46]. However, it would be an underestimation to consider only the pro-inflammatory characteristics of Group 1 macrophages. Gene expression profile analysis showed that the anti-inflammatory cytokine *Il10* is up-regulated in Group 1 BMDMs (Table S2 and Figure 2) and, accordingly, GSEA analysis revealed that *Il10* signaling is enriched in these immune cells (Figure 3). The simultaneous expression of pro- and anti-inflammatory markers might be induced by several mechanisms but, based on our microarray data, we can infer some insights. For example, we observed that miR155 and mir146 are present in the list of differentially regulated transcripts (Table S2). These miRNAs are reported to play an important role in modulating macrophage phenotype [47]. Indeed, miR155 and miR146 were described to promote M1 and M2 polarization, respectively [48,49]. Since both were found to be up-regulated in Group 1 macrophages, this finding may corroborate the idea of the co-existence of M1 and M2 features in Group 1 macrophages. Moreover, miR223, down-modulated in Group 1 BMDMs, is described to limit inflammation and to drive macrophages towards an M2 phenotype [50]. Interestingly, miR223 is regulated by *Pparg* [51], which is also down-regulated in Group 1 compared to Group 2 macrophages (Table S2 and Figure 2).

Therefore, Group 1 macrophages show two opposite natures that collaborate to create a favorable background for HPD development. 

We previously reported that the infiltration of a particular subset of macrophages co-expressing CD33, PD-L1 and CD163 was associated with HPD occurrence in NSCLC patients [3]. Interestingly, when a metagene based on the aforementioned markers was applied to our microarray profile data, the highest metagene score was found in Group 1 macrophages compared to all the other experimental groups. Interestingly, hierarchical clustering, performed using only those three genes, showed clear and independent segregation of Group 1 BMDMs. These in vitro findings not only sustain what we observed in the human setting but also strengthen the translational value of the present work. 

### 3.2. Potential Consequences of Anti-PD1 Antibody Treatment on Macrophage Harboring “HPD-Related Phenotype”

Another open question is how this peculiar macrophage phenotype can be associated with HPD onset. Indeed, the molecular events occurring in macrophages upon interaction with the anti-PD1 antibody are still to be completely elucidated but the findings presented in this study and our previously published data open up two possible scenarios. PD1 is a receptor that inhibits not only T lymphocytes but also macrophage activation and function [52]. Gordon et al. reported that the blockade of macrophage PD1 is able to awaken these immune cells from a suppressive condition and restore macrophage anti-tumor activity [53]. In our specific context, it may be possible that disrupting this inhibitory signal may further exacerbate the pro-inflammatory nature of Group 1 macrophages, thus increasing the risk of a local and systemic severe inflammatory response (i.e., cytokine storm) that may lead to life-threatening adverse effects [54]. However, our results, showing enrichment of the “Common UP in Group 1” gene set in untreated compared to anti-PD1-treated H460 xenografts, probably suggest that anti-PD1 antibody administration further impacts macrophage phenotype without exacerbating the pro-inflammatory nature of these immune cells. Another possibility may emerge from GSEA analysis. We found an enrichment of the TLR-related signaling pathway in Group 1 macrophages and TLR can represent a double-edged sword in the context of cancer [55]. We previously reported that macrophages treated with a TLR9 agonist in combination with an anti-PD1 antibody acquired a phenotype characterized by immunosuppressive and pro-tumor activity, as demonstrated in vivo and in vitro [56]. We also demonstrated that this effect is mediated by the anti-PD1 antibody Fc domain [56]. We speculated that the binding of an anti-PD1 Fc portion to macrophage Fc receptors in combination with TLR stimulation determined the generation of M2b macrophages, a subtype of regulatory/immunosuppressive macrophages secreting both pro- and anti-inflammatory cytokines and associated with cancer progression [57]. It is plausible to hypothesize that the anti-PD1 antibody, through its Fc domain, may also lead to a switch towards an M2b phenotype in Group 1 macrophages where TLR signaling was already activated by molecules secreted by tumor cells. Finally, we performed a series of experiments aimed at understanding the chemical nature of the molecule(s) involved in the described effect on the macrophage transcriptome. First of all, we determined that heat-inactivated H460 CM has a strongly reduced stimulatory capability on BMDMs compared to naïve CM. On the contrary, the *Lcn2* mRNA level appears to be increased by heated CM (Figure 6). Recently, it was described that *LCN2* expression is regulated by *NUPR1* [25], a stress-inducible protein [58], found to be up-regulated in Group 1 versus Group 2 BMDMs (Table S2) and by heated CM (Figure S1). Since *Lcn2* acts as an anti-inflammatory regulator of macrophage activation [59], it is possible to hypothesize that Group 1 macrophages, characterized by strong pro-inflammatory features, may be subjected to potent cellular stress that may be counterbalanced by the transcription of stress-inducible genes, such as *Nupr1* that, in turn, promotes the up-regulation of anti-inflammatory molecules as *Lcn2*. Data obtained with heated CMs may also raise the possibility that free proteins contained in the CM can, at least in part, directly participate in macrophage polarization. However, subsequent investigations made this scenario unlikely. Indeed, lipid removal completely nullified any effect mediated by CM on macrophages and the exposure of BMDMs to CM-derived EVs reproduced what was observed with untouched CM. This evidence indicates that EVs represent the main character in the process of macrophage reprogramming by activating multiple cellular pathways including TLR signaling. This finding perfectly fits with previous reports indicating that EVs, released by tumor cells, are able to induce a pro-inflammatory phenotype in macrophages via TLR signaling engagement and influence their phenotype [60,61]. Moreover, the identification of EVs as major players in tumor-elicited macrophage polarization can also explain the data obtained using heat-inactivated CM. Elevated temperatures can decrease EV concentration and alter their physicochemical properties [62] and, therefore, it is highly possible that heat-inactivated CM did not contain sufficient EVs to produce any impact on BMDMs and/or these EVs were not functional.

## 4. Materials and Methods

### 4.1. Cell Lines and Culture Conditions

The human non-small cell lung cancer (NSCLC) cell lines NCI-H460 (H460, HTB-177™), A549 (CCL-185™), NCI-H1299 (H1299, CRL-5803™), Calu-1 (HTB-54™), THP-1 human monocyte cell line (TIB-202™) and RAW264.7 mouse macrophage cell line (TIB-71™) were purchased from ATCC (American Type Culture Collection, Manassas, VA, USA). PC9 (formerly known as PC-14) was purchased from ECACC General Cell Collection (Salisbury, UK). Cell lines were authenticated by STR analysis at Fondazione IRCCS–Istituto Nazionale dei Tumori (Milan, Italy) using GenePrint 10 System (Promega, Madison, WI, USA). NSCLC cell lines and THP-1 cells were maintained in RPMI 1640 with L-glutamine (Gibco, Thermo Fisher Scientific, Waltham, MA, USA) supplemented with 10% fetal bovine serum (FBS, Gibco, Thermo Fisher Scientific, Waltham, MA, USA) at 37 °C in a 5% CO_2_ atmosphere. RAW264.7 cell line was maintained in DMEM with L-glutamine (Gibco, Thermo Fisher Scientific, Waltham, MA, USA) supplemented with 10% FBS (Gibco, Thermo Fisher Scientific, Waltham, MA, USA) at 37 °C in a 5% CO_2_ atmosphere. Cultures were regularly tested for Mycoplasma by using the mycoAlert Plus Kit (Lonza Group, Basel, Switzerland).

### 4.2. Preparation of NSCLC Cell Line Conditioned Medium (CMs)

NSCLC cell lines were seeded in 6-well plates (Thermo Fisher Scientific, Waltham, MA, USA) at a density of 1 × 10^6^ cells/well in complete medium (RPMI 1640 with L-glutamine supplemented with 10% FBS) (Gibco, Thermo Fisher Scientific, Waltham, MA, USA). The following day, medium was replaced with 2 mL of fresh serum-free medium and cells were cultured for additional 24 h. CMs were collected under sterile conditions, centrifuged at 1500 rpm for 10 min at Room Temperature (RT). To denature proteins in tumor CM (heated-CM), H460-CM was heated at 95 °C for 10 min in a Thermomixer Comfort (Eppendorf SE, Hamburg, Germany), followed by centrifugation at 13,000 rpm for 20 min at RT. Lipid content was removed from H460-CM (lipid removed-CM), obtained as above, using Cleanascite™ Lipid Removal Reagent and Clarification (www.biotechsupportgroup.com/Cleanascite-Lipid-Removal-Reagent-p/x2555.htm accessed on 20 July 2022), according to the manufacturer’s instructions (Biotech Support Group, Monmouth Junction, NJ, USA). Non-conditioned control medium, serum-free RPMI 1640 medium without cell incubation, was prepared with the same protocol as the CMs and served for the treatment of control macrophages. CMs were immediately collected and utilized for macrophage treatment.

### 4.3. Gene Expression Profile 

BMDMs, generated as previously described [56], were seeded in 12-well plates (Thermo Fisher Scientific, Waltham, MA, USA) at a density of 8 × 10^5^ cells/well in Iscove’s Modified Dulbecco’s Medium (IMDM, Gibco, Thermo Fisher Scientific, Waltham, MA, USA) supplemented with 10% FBS (Gibco, Thermo Fisher Scientific, Waltham, MA, USA) and 1% penicillin/streptomycin solution (Thermo Fisher Scientific, Waltham, MA, USA). The following day, culture medium was removed and BMDMs were exposed to 2 mL of the different NSCLC CMs for 24 h. For cytokine-induced macrophage polarization, BMDMs were incubated for 24 h with 2 mL of serum-free medium containing 10 ng/mL lipopolysaccharide (LPS, Merck KGaA, Darmstadt, Germany) plus 20 ng/mL of IFN-γ (Peprotech, Cranbury, NJ, USA) or with 20 ng/mL IL-4 (Peprotech) to obtain classically activated (M1) or alternatively activated (M2) macrophages, respectively [4]. Macrophages receiving fresh plain serum-free medium were considered as M0. The animal experiments were authorized by the Institutional Animal Welfare Body and the Italian Ministry of Health and performed in accordance with national law (D.lgs 26/2014) and the Guidelines for the Welfare of Animals in Experimental Neoplasia [63]. At the end of treatment, BMDMs mRNA was isolated using Direct-zol™ RNA MicroPrep (Zymo Research, Irvine, CA, USA) according to the manufacturer’s instructions. mRNA quality check and quantification were carried out by 4200TapeStation (Agilent Technologies, Santa Clara, CA, USA) and Qubit Fluorometer (Thermo Fisher Scientific, Waltham, MA, USA), respectively. RNA expression was assessed using the Clariom™ D Assay, mouse (Thermo Fisher Scientific, Waltham, MA, USA) following manufacturer’s protocols. 

H460 tumor xenografts grown in athymic nude mice were collected from the in vivo experiment published in [3]. After sacrifice, mouse tumors were excised, rinsed in cold PBS and immediately snap-frozen in liquid nitrogen until RNA extraction. Solid tumor masses were pulverized using a Mikrodismembrator (B. Braun Biotech International GmbH, Melsungen, Germany). Total RNA was extracted with QIAzol Lysis Reagent (Qiagen Redwood City, CA, USA) and RNeasy kit (Qiagen) according to the manufacturer’s instructions, processed and hybridized to Illumina MouseWG-6 v2.0 expression beadchip (Illumina, Inc., San Diego, CA, USA) according to the manufacturer’s instructions. Primary data were collected using the supplied scanner software and analyzed using BeadStudio Version 3 software (Illumina, Inc., San Diego, CA, USA). Intensity values of each hybridization were quality-checked and the dataset was normalized using the quantile algorithm. Microarray dataset and sample information are deposited in the Gene Expression Omnibus database (https://www.ncbi.nlm.nih.gov/gds accessed on 4 August 2022) under the accession numbers GSE210340 (gene profiling of M0, M1 and M2 BMDMs and those incubated with NSCLC cell line CMs) and GSE210547 (gene expression data of H460 tumor xenografts untreated and anti-PD1 antibody treated).

### 4.4. Bioinformatic Analysis

Raw data from Clariom™ D Assay.CEL files were imported in the Transcriptomic Analysis Console (TAC, version 4.0.2.15, Thermo Fisher Scientific, Waltham, MA, USA) and normalized using the robust multichip average (RMA) algorithm. Principal Component Analysis (PCA) analysis and volcano plot were generated by TAC software (Thermo Fisher Scientific, Waltham, MA, USA). Comparisons between the different experimental groups were carried out by TAC software (Thermo Fisher Scientific, Waltham, MA, USA). Annotated transcripts were considered differentially expressed (DEGs) if they showed a False Discovery Rate (FDR) < 0.01 and a Fold-Change (FC) ≤ −2 and ≥2 (Tables S2 and S3). Venn diagrams showing the intersection of up- and down-modulated DEGs in Group 1 versus every other group were performed utilizing a web-based Venn diagram generator (https://bioinformatics.psb.ugent.be/webtools/Venn/ accessed on 22 September 2022). Genes were always up- or down-regulated in every comparison and were utilized to generate two gene sets, named “Common UP in Group 1” and “Common DOWN in Group 1” (Table S3). The online gene-list analysis tool Metascape was utilized to identify biological functions and processes associated with “Common UP in Group 1” and “Common DOWN in Group 1” gene lists. Metascape analysis was carried out using default parameters.

Pathway-based analysis was performed by interrogating the Core Analysis function of the Ingenuity Pathway Analysis (IPA) software (Qiagen). Enrichment analysis was performed using Gene Set Enrichment Analysis (GSEA) Desktop Application v4.2.3.0 (http://www.broadinstitute.org/gsea/index.jsp accessed on 1 August 2022) [10] with the Reactome collection (v7.5.1 accessed on 1 August 2022), containing 1615 gene sets, retrieved from the molecular signature database (https://www.gsea-msigdb.org/gsea/msigdb/genesets.jsp?collection=CP:REACTOME accessed on 1 August 2022). Pathways with FDR *q* value < 0.05 were considered statistically significant. Single sample GSEA (ssGSEA) analysis (version 10.0.11), a strategy to calculate enrichment scores for each pairing of a sample and gene set, was performed using the specific tool incorporated in the genomic analysis platform GenePattern (https://cloud.genepattern.org/ accessed on 12 July 2022) [64]. Gene expression data from a publicly available dataset containing transcriptomic data of macrophages polarized toward different phenotypes were retrieved from Gene Expression Omnibus (GEO) website (GSE32690). Data were imported in the ssGSEA suite present in GenePattern platform, normalized by rank and ssGSEA was performed. “HPD-related metagene” was constructed by averaging the log_2_ normalized expression values of the *Cd33*, *PD-L1* (*Cd274*) and *Cd163* genes for each sample. Hierarchical clustering and heatmaps on normalized genomic data were obtained using the appropriate suites present in the GenePattern platform.

### 4.5. Extracellular Vesicles (EVs) Isolation

EVs from H460 cells CM were purified by differential centrifugation process. Specifically, 1 × 10^6^ cells/well were seeded in 6-well plates (Thermo Fisher Scientific Inc.) in complete medium (RPMI 1640 with L-glutamine supplemented with 10% FBS). The day after cell seeding, medium was replaced with 2 mL of fresh serum-free medium and cells were cultured for additional 24 h. H460-CM from different wells was collected and pooled and the volume was recorded. CM was then centrifuged at 300× *g* for 10 min and, subsequently, at 3200× *g* for 25 min to remove residual cells and debris. To exclude large vesicles, the supernatant was filtered through 0.22 μm filters (Millipore, Burlington, MA, USA) and then ultracentrifuged at 120,000× *g* for 90 min at 4 °C using a TLA-100.3 fixed-angle rotor in a TL-100 ultracentrifuge (Beckman Coulter, Brea, CA, USA). The resulting supernatant was collected as CM-EV-depleted medium (EV-free medium) and served for the treatment of control BMDMs. EV-enriched pellet was washed in phosphate-buffered saline (PBS, Gibco, Thermo Fisher Scientific, Waltham, MA, USA) at the same ultracentrifuge speed for 60 min at 4 °C. The pellet was finally resuspended in different buffers depending on the subsequent analysis. For EVs’ characterization, EV pellet was resuspended in PBS (Gibco, Thermo Fisher Scientific, Waltham, MA, USA) and concentration and size distribution were determined by NanoSight NS300 instrument (Malvern Panalytical, Malvern, UK). Five 30 s videos were recorded for each sample with a camera level set at 15/16 and a detection threshold set between 2 and 7. The videos were subsequently analyzed with NTA 3.2 software (Malvern Panalytical) to calculate the size and concentration of the particles. Auto settings were used for the analysis. 

### 4.6. Western Blotting

EV pellets were directly lysed in RIPA buffer (Merck KGaA, Darmstadt, Germany) with protease and phosphatase inhibitor cocktail (Merck KGaA, Darmstadt, Germany) and stored at −80 °C until use. The protein content of the purified EVs was determined by using the Bradford assay (Bio-Rad, Hercules, CA, USA). Then, protein lysate (40 μg) was loaded on a Bolt 4–12% Bis-Tris gel (Thermo Fisher Scientific, Waltham, MA, USA). Western blot analyses were performed using the following antibodies: anti-CD9 (Cell Signaling Technology, Danvers, MA, USA. Dilution: 1:1000), anti-CD81 (Cell Signaling Technology, Danvers, MA, USA. Dilution: 1:1000) primary antibodies and the corresponding anti-mouse and anti-rabbit peroxidase-linked secondary antibodies (Cell Signaling Technology, Danvers, MA, USA. Dilution: 1:5000). Signal detection was performed via chemiluminescence reaction (ECL, GE Healthcare, Chicago, IL, USA) using the MINI HD9 Western Blot Imaging System (Cleaver Scientific Ltd., Rugby, UK).

### 4.7. Transmission Electron Microscopy (TEM)

Transmission electron microscopy (TEM) was performed to evaluate the morphological characteristics of the EVs isolated from the CM and their release by H460 cell line. The EVs pellet was resuspended in 0.2 M sodium cacodylate buffer (Merck KGaA, Darmstadt, Germany). For the analysis of isolated EVs, 5 μL of EVs samples were placed on formvar carbon-coated copper grids. After 3 min, the excess of aqueous solution was removed with filter paper and grids were air dried. Cellular ultrastructure analysis was performed on cells grown to near confluence on Petri dish and fixed as previously described [65]. Next, the 2D monolayer cultures in situ on Petri dishes were post-fixed with 1% osmium tetroxide at 0 °C o/n, stained with 2% aqueous uranyl acetate, dehydrated with ascending concentration of ethanol at 4 °C and embedded in Epon–Araldite resin. Ultrathin sections were obtained with a Leica Supernova ultramicrotome (Reichert Ultracut, Wien, Austria) and counterstained with lead citrate. Grids were observed with a Zeiss EM10 electron microscope (Carl Zeiss, Oberkochen, Germany) and all measurements were manually performed using the Image Pro-Plus software (version 6.0) (Media Cybernetics, Inc., Washington, WA, USA) on randomly acquired micrographs.

### 4.8. In Vitro Studies 

To evaluate the chemical nature of the molecules present in the CMs able to stimulate macrophages, BMDMs were seeded in 12-well plates (Thermo Fisher Scientific, Waltham, MA, USA) at a density of 8 × 10^5^ cells/well in Iscove’s Modified Dulbecco’s Medium (IMDM, Gibco, Thermo Fisher Scientific, Waltham, MA, USA) supplemented with 10% FBS (Gibco, Thermo Fisher Scientific, Waltham, MA, USA) and 1% penicillin/streptomycin solution (Thermo Fisher Scientific, Waltham, MA, USA). The following day, culture medium was removed and BMDMs incubated with 2 mL of heated or lipid-removed CMs, as described above. Non-conditioned control medium, serum-free RPMI 1640 medium without cell incubation, was prepared with the same protocol as the CMs and served for the treatment of control BMDMs. In order to expose macrophages to the same quantity of EVs present in 2 mL of CMs, EV pellet was resuspended in serum-free medium in a volume equal to the initial volume of collected supernatants. EVs resuspended in 2 mL of serum-free medium were then utilized for macrophage stimulation for 24 h. Control BMDMs received EVs-free medium. RAW264.7 mouse macrophage cells were seeded in 12-well plates (Thermo Fisher Scientific, Waltham, MA, USA) at a density of 8 × 10^5^ cells/well. The day after cell seeding, RAW264.7 were exposed to H460-, PC9- or H1299-derived EVs, as described above. THP-1-derived human macrophages, obtained as detailed in [66], were seeded in 12-well plates (Thermo Fisher Scientific, Waltham, MA, USA) at a density of 3 × 10^5^ cells/mL and then treated with the same protocol utilized for RAW264.7 cell line. At the end of each treatment, mRNA was extracted using Direct-zol™ RNA MicroPrep (Zymo Research, Irvine, CA, USA) according to the manufacturer’s instructions and immediately store at −80 °C until use.

### 4.9. Real-Time PCR 

Real-time PCR was performed on the same RNA samples utilized for microarray analysis and on the RNA extracted from BMDMs, RAW264.7 cells or THP-1-derived macrophages after different in vitro treatments. In all cases, High-Capacity RNA-to-cDNA Kit (Applied Biosystems, Thermo Fisher Scientific, Waltham, MA, USA) was utilized for mRNA reverse transcription. Real-time PCR analysis was carried out using TaqMan^®^ Fast Universal PCR Master Mix (Applied Biosystems, Thermo Fisher Scientific, Waltham, MA, USA) on a StepOne Real-Time PCR System (Applied Biosystems, Thermo Fisher Scientific, Waltham, MA, USA). The complete list of TaqMan^®^ gene expression assays (Applied Biosystems, Thermo Fisher Scientific, Waltham, MA, USA) utilized for real-time PCR analysis is provided in Table S6. Each gene mRNA level was normalized to the appropriate housekeeping expression. PCR data were analyzed by the 2^−ΔCt^ or by 2^−ΔΔCt^ method.

### 4.10. Statistical Analysis 

GraphPad Prism (GraphPad Software, San Diego, CA, USA) was utilized as statistical software. Before performing every statistical analysis, normal distribution of data was verified by normality tests. Differences between two groups were determined by two-tailed unpaired Student’s *t*-test or the Mann–Whitney U test for nonparametric data. Comparisons among > 2 groups were performed by one-way ANOVA followed by Tukey’s multiple comparison test with a single pooled variance or by the Kruskal–Wallis test followed by Dunn’s multiple comparison test for nonparametric data. Data are represented as mean ± standard error (mean ± SEM) and differences were considered significant at *p* < 0.05.

## 5. Conclusions

The present study, unraveling the contribution of EVs by inducing an HPD-mediated phenotype on macrophages, contributes to a better comprehension of the cellular and molecular mechanisms underlying this detrimental clinical phenomenon. EVs may represent either a new diagnostic tool to identify patients that should not be treated with anti-PD1/PDL1 immunotherapy and a new potential therapeutic target to reverse HPD. Further studies will be needed to precisely identify which factor(s), constituting the EV cargo, is/are responsible for the observed effect on macrophages.

## Data Availability

The datasets generated during the current study are available in the Gene Expression Omnibus repository (https://www.ncbi.nlm.nih.gov/gds accessed on 4 August 2022) under the accession numbers GSE210340 and GSE210547.

## Supplementary Materials

The following supporting information can be downloaded at: https://www.mdpi.com/article/10.3390/ijms232415802/s1, Table S1: Mutational landscape of NSCLC cell lines; Table S2: List of differentially expressed transcripts between Group 1 and Group 2 BMDMs; Table S3: Lists of differentially expressed annotated transcripts up- or down-modulated in Group 1 macrophages compared to all the other experimental groups; Table S4: “Common UP in Group 1” and “Common DOWN in Group 1” gene lists; Table S5: Size distribution analysis of H460-EVs. Table S6: List of TaqMan^®^ gene expression assays utilized for real-time PCR analysis; Figure S1: mRNA expression level of cytokines and chemokines in BMDMs exposed to NSCLC cell line-conditioned medium; Figure S2: Effect of protein and lipid removal from H460 CM on *Nupr1* and *Trib3* expression; Figure S3: Effect of H460-, PC9- and H1299-EVs on RAW264.7 mouse macrophage cells. Figure S4: Effect of H460-, PC9- and H1299-EVs on human THP-1-derived macrophages. Supplementary Legends: Supplementary table and figure legends.

## References

[1] Champiat S., Dercle L., Ammari S., Massard C., Hollebecque A., Postel-Vinay S., Chaput N., Eggermont A., Marabelle A., Soria J.-C. (2017). Hyperprogressive Disease Is a New Pattern of Progression in Cancer Patients Treated by Anti-PD-1/PD-L1. Clin. Cancer Res..

[2] Camelliti S., Le Noci V., Bianchi F., Moscheni C., Arnaboldi F., Gagliano N., Balsari A., Garassino M.C., Tagliabue E., Sfondrini L. (2020). Mechanisms of hyperprogressive disease after immune checkpoint inhibitor therapy: What we (don’t) know. J. Exp. Clin. Cancer Res..

[3] Lo Russo G., Moro M., Sommariva M., Cancila V., Boeri M., Centonze G., Ferro S., Ganzinelli M., Gasparini P., Huber V. (2019). Antibody-Fc/FcR Interaction on Macrophages as a Mechanism for Hyperprogressive Disease in Non-small Cell Lung Cancer Subsequent to PD-1/PD-L1 Blockade. Clin. Cancer Res..

[4] Zhou J., Tang Z., Gao S., Li C., Feng Y., Zhou X. (2020). Tumor-Associated Macrophages: Recent Insights and Therapies. Front. Oncol..

[5] Wang H., Yung M.M.H., Ngan H.Y.S., Chan K.K.L., Chan D.W. (2021). The Impact of the Tumor Microenvironment on Macrophage Polarization in Cancer Metastatic Progression. Int. J. Mol. Sci..

[6] Rodak O., Peris-Díaz M.D., Olbromski M., Podhorska-Okołów M., Dzięgiel P. (2021). Current Landscape of Non-Small Cell Lung Cancer: Epidemiology, Histological Classification, Targeted Therapies, and Immunotherapy. Cancers.

[7] Kumar S., Ghosh S., Sharma G., Wang Z., Kehry M.R., Marino M.H., Neben T.Y., Lu S., Luo S., Roberts S. (2021). Preclinical characterization of dostarlimab, a therapeutic anti-PD-1 antibody with potent activity to enhance immune function in in vitro cellular assays and in vivo animal models. mAbs.

[8] Moon E.K., Ranganathan R., Eruslanov E., Kim S., Newick K., O’Brien S., Lo A., Liu X., Zhao Y., Albelda S.M. (2016). Blockade of Programmed Death 1 Augments the Ability of Human T Cells Engineered to Target NY-ESO-1 to Control Tumor Growth after Adoptive Transfer. Clin. Cancer Res..

[9] Caiola E., Iezzi A., Tomanelli M., Bonaldi E., Scagliotti A., Colombo M., Guffanti F., Micotti E., Garassino M.C., Minoli L. (2020). LKB1 Deficiency Renders NSCLC Cells Sensitive to ERK Inhibitors. J. Thorac. Oncol..

[10] Sun L., Liu X., Fu H., Zhou W., Zhong D. (2016). 2-Deoxyglucose Suppresses ERK Phosphorylation in LKB1 and Ras Wild-Type Non-Small Cell Lung Cancer Cells. PLoS ONE.

[11] Yang C.-H., Chou H.-C., Fu Y.-N., Yeh C.-L., Cheng H.-W., Chang I.-C., Liu K.-J., Chang G.-C., Tsai T.-F., Tsai S.-F. (2015). EGFR over-expression in non-small cell lung cancers harboring EGFR mutations is associated with marked down-regulation of CD82. Biochim. Biophys. Acta.

[12] Koivunen J.P., Kim J., Lee J., Rogers A.M., Park J.O., Zhao X., Naoki K., Okamoto I., Nakagawa K., Yeap B.Y. (2008). Mutations in the LKB1 tumour suppressor are frequently detected in tumours from Caucasian but not Asian lung cancer patients. Br. J. Cancer.

[13] Litmanovich A., Khazim K., Cohen I. (2018). The Role of Interleukin-1 in the Pathogenesis of Cancer and its Potential as a Therapeutic Target in Clinical Practice. Oncol. Ther..

[14] Hirano T. (2021). IL-6 in inflammation, autoimmunity and cancer. Int. Immunol..

[15] La Fleur L., Boura V.F., Alexeyenko A., Berglund A., Pontén V., Mattsson J.S.M., Djureinovic D., Persson J., Brunnström H., Isaksson J. (2018). Expression of scavenger receptor MARCO defines a targetable tumor-associated macrophage subset in non-small cell lung cancer. Int. J. Cancer.

[16] Marzio R., Mauël J., Betz-Corradin S. (1999). CD69 and regulation of the immune function. Immunopharmacol. Immunotoxicol..

[17] Jung M., Ören B., Mora J., Mertens C., Dziumbla S., Popp R., Weigert A., Grossmann N., Fleming I., Brüne B. (2016). Lipocalin 2 from macrophages stimulated by tumor cell-derived sphingosine 1-phosphate promotes lymphangiogenesis and tumor metastasis. Sci. Signal..

[18] Schaefer L., Babelova A., Kiss E., Hausser H.-J., Baliova M., Krzyzankova M., Marsche G., Young M.F., Mihalik D., Götte M. (2005). The matrix component biglycan is proinflammatory and signals through Toll-like receptors 4 and 2 in macrophages. J. Clin. Investig..

[19] Luikart S., Wahl D., Hinkel T., Masri M., Oegema T. (1999). A fragment of alpha-actinin promotes monocyte/macrophage maturation in vitro. Exp. Hematol..

[20] Liu Q., Zhao E., Geng B., Gao S., Yu H., He X., Li X., Dong G., You B. (2022). Tumor-associated macrophage-derived exosomes transmitting miR-193a-5p promote the progression of renal cell carcinoma via TIMP2-dependent vasculogenic mimicry. Cell Death Dis..

[21] Ip W.K.E., Hoshi N., Shouval D.S., Snapper S., Medzhitov R. (2017). Anti-inflammatory effect of IL-10 mediated by metabolic reprogramming of macrophages. Science.

[22] Tian X., Xie G., Xiao H., Ding F., Bao W., Zhang M. (2019). CXCR4 knockdown prevents inflammatory cytokine expression in macrophages by suppressing activation of MAPK and NF-κB signaling pathways. Cell Biosci..

[23] Subramanian A., Kuehn H., Gould J., Tamayo P., Mesirov J.P. (2007). GSEA-P: A desktop application for Gene Set Enrichment Analysis. Bioinformatics.

[24] Riquelme P., Tomiuk S., Kammler A., Fändrich F., Schlitt H.J., Geissler E.K., Hutchinson J.A. (2013). IFN-γ-induced iNOS expression in mouse regulatory macrophages prolongs allograft survival in fully immunocompetent recipients. Mol. Ther..

[25] Liu J., Song X., Kuang F., Zhang Q., Xie Y., Kang R., Kroemer G., Tang D. (2021). NUPR1 is a critical repressor of ferroptosis. Nat. Commun..

[26] Huang C., Santofimia-Castaño P., Iovanna J. (2021). NUPR1: A Critical Regulator of the Antioxidant System. Cancers.

[27] Reed T., Schorey J., D’Souza-Schorey C. (2021). Tumor-Derived Extracellular Vesicles: A Means of Co-opting Macrophage Polarization in the Tumor Microenvironment. Front. Cell Dev. Biol..

[28] Kalluri R., LeBleu V.S. (2020). The biology, function, and biomedical applications of exosomes. Science.

[29] Park J.V., Chandra R., Cai L., Ganguly D., Li H., Toombs J.E., Girard L., Brekken R.A., Minna J.D. (2022). Tumor Cells Modulate Macrophage Phenotype in a Novel In Vitro Co-Culture Model of the NSCLC Tumor Microenvironment. J. Thorac. Oncol..

[30] Kelley N., Jeltema D., Duan Y., He Y. (2019). The NLRP3 Inflammasome: An Overview of Mechanisms of Activation and Regulation. Int. J. Mol. Sci..

[31] Theivanthiran B., Yarla N., Haykal T., Nguyen Y.-V., Cao L., Ferreira M., Holtzhausen A., Al-Rohil R., Salama A.K.S., Beasley G.M. (2022). Tumor-intrinsic NLRP3-HSP70-TLR4 axis drives premetastatic niche development and hyperprogression during anti-PD-1 immunotherapy. Sci. Transl. Med..

[32] Pan Y., Yu Y., Wang X., Zhang T. (2020). Tumor-Associated Macrophages in Tumor Immunity. Front. Immunol..

[33] Greten F.R., Grivennikov S.I. (2019). Inflammation and Cancer: Triggers, Mechanisms, and Consequences. Immunity.

[34] Garon E.B., Chih-Hsin Yang J., Dubinett S.M. (2020). The Role of Interleukin 1β in the Pathogenesis of Lung Cancer. JTO Clin. Res. Rep..

[35] Zhang J., Veeramachaneni N. (2022). Targeting interleukin-1β and inflammation in lung cancer. Biomark. Res..

[36] Liu C., Yang L., Xu H., Zheng S., Wang Z., Wang S., Yang Y., Zhang S., Feng X., Sun N. (2022). Systematic analysis of IL-6 as a predictive biomarker and desensitizer of immunotherapy responses in patients with non-small cell lung cancer. BMC Med..

[37] Silva E.M., Mariano V.S., Pastrez P.R.A., Pinto M.C., Castro A.G., Syrjanen K.J., Longatto-Filho A. (2017). High systemic IL-6 is associated with worse prognosis in patients with non-small cell lung cancer. PLoS ONE.

[38] Montfort A., Colacios C., Levade T., Andrieu-Abadie N., Meyer N., Ségui B. (2019). The TNF Paradox in Cancer Progression and Immunotherapy. Front. Immunol..

[39] Allavena P., Digifico E., Belgiovine C. (2021). Macrophages and cancer stem cells: A malevolent alliance. Mol. Med..

[40] Kiss M., Vande Walle L., Saavedra P.H.V., Lebegge E., van Damme H., Murgaski A., Qian J., Ehling M., Pretto S., Bolli E. (2021). IL1β Promotes Immune Suppression in the Tumor Microenvironment Independent of the Inflammasome and Gasdermin D. Cancer Immunol. Res..

[41] Caetano M.S., Zhang H., Cumpian A.M., Gong L., Unver N., Ostrin E.J., Daliri S., Chang S.H., Ochoa C.E., Hanash S. (2016). IL6 Blockade Reprograms the Lung Tumor Microenvironment to Limit the Development and Progression of K-ras-Mutant Lung Cancer. Cancer Res..

[42] Salomon B.L., Leclerc M., Tosello J., Ronin E., Piaggio E., Cohen J.L. (2018). Tumor Necrosis Factor α and Regulatory T Cells in Oncoimmunology. Front. Immunol..

[43] Tammaro A., Derive M., Gibot S., Leemans J.C., Florquin S., Dessing M.C. (2017). TREM-1 and its potential ligands in non-infectious diseases: From biology to clinical perspectives. Pharmacol. Ther..

[44] Raggi F., Bosco M.C. (2020). Targeting Mononuclear Phagocyte Receptors in Cancer Immunotherapy: New Perspectives of the Triggering Receptor Expressed on Myeloid Cells (TREM-1). Cancers.

[45] Ford J.W., Gonzalez-Cotto M., MacFarlane A.W., Peri S., Howard O.M.Z., Subleski J.J., Ruth K.J., Haseebuddin M., Al-Saleem T., Yang Y. (2021). Tumor-Infiltrating Myeloid Cells Co-Express TREM1 and TREM2 and Elevated TREM-1 Associates With Disease Progression in Renal Cell Carcinoma. Front. Oncol..

[46] Wu Q., Zhou W., Yin S., Zhou Y., Chen T., Qian J., Su R., Hong L., Lu H., Zhang F. (2019). Blocking Triggering Receptor Expressed on Myeloid Cells-1-Positive Tumor-Associated Macrophages Induced by Hypoxia Reverses Immunosuppression and Anti-Programmed Cell Death Ligand 1 Resistance in Liver Cancer. Hepatology.

[47] Essandoh K., Li Y., Huo J., Fan G.-C. (2016). MiRNA-Mediated Macrophage Polarization and its Potential Role in the Regulation of Inflammatory Response. Shock.

[48] Pasca S., Jurj A., Petrushev B., Tomuleasa C., Matei D. (2020). MicroRNA-155 Implication in M1 Polarization and the Impact in Inflammatory Diseases. Front. Immunol..

[49] Peng X., He F., Mao Y., Lin Y., Fang J., Chen Y., Sun Z., Zhuo Y., Jiang J. (2022). miR-146a promotes M2 macrophage polarization and accelerates diabetic wound healing by inhibiting the TLR4/NF-κB axis. J. Mol. Endocrinol..

[50] Dang C.P., Leelahavanichkul A. (2020). Over-expression of miR-223 induces M2 macrophage through glycolysis alteration and attenuates LPS-induced sepsis mouse model, the cell-based therapy in sepsis. PLoS ONE.

[51] Ying W., Tseng A., Chang R.C.-A., Morin A., Brehm T., Triff K., Nair V., Zhuang G., Song H., Kanameni S. (2015). MicroRNA-223 is a crucial mediator of PPARγ-regulated alternative macrophage activation. J. Clin. Investig..

[52] Lu D., Ni Z., Liu X., Feng S., Dong X., Shi X., Zhai J., Mai S., Jiang J., Wang Z. (2019). Beyond T Cells: Understanding the Role of PD-1/PD-L1 in Tumor-Associated Macrophages. J. Immunol. Res..

[53] Gordon S.R., Maute R.L., Dulken B.W., Hutter G., George B.M., McCracken M.N., Gupta R., Tsai J.M., Sinha R., Corey D. (2017). PD-1 expression by tumour-associated macrophages inhibits phagocytosis and tumour immunity. Nature.

[54] Tay S.H., Toh M.M.X., Thian Y.L., Vellayappan B.A., Fairhurst A.-M., Chan Y.H., Aminkeng F., Bharwani L.D., Huang Y., Mak A. (2022). Cytokine Release Syndrome in Cancer Patients Receiving Immune Checkpoint Inhibitors: A Case Series of 25 Patients and Review of the Literature. Front. Immunol..

[55] Le Noci V., Bernardo G., Bianchi F., Tagliabue E., Sommariva M., Sfondrini L. (2021). Toll Like Receptors as Sensors of the Tumor Microbial Dysbiosis: Implications in Cancer Progression. Front. Cell Dev. Biol..

[56] Camelliti S., Le Noci V., Bianchi F., Storti C., Arnaboldi F., Cataldo A., Indino S., Jachetti E., Figini M., Colombo M.P. (2021). Macrophages Impair TLR9 Agonist Antitumor Activity through Interacting with the Anti-PD-1 Antibody Fc Domain. Cancers.

[57] Wang L.-X., Zhang S.-X., Wu H.-J., Rong X.-L., Guo J. (2019). M2b macrophage polarization and its roles in diseases. J. Leukoc. Biol..

[58] Martin T.A., Li A.X., Sanders A.J., Ye L., Frewer K., Hargest R., Jiang W.G. (2021). NUPR1 and its potential role in cancer and pathological conditions (Review). Int. J. Oncol..

[59] Warszawska J.M., Gawish R., Sharif O., Sigel S., Doninger B., Lakovits K., Mesteri I., Nairz M., Boon L., Spiel A. (2013). Lipocalin 2 deactivates macrophages and worsens pneumococcal pneumonia outcomes. J. Clin. Investig..

[60] Han C., Zhang C., Wang H., Zhao L. (2021). Exosome-mediated communication between tumor cells and tumor-associated macrophages: Implications for tumor microenvironment. Oncoimmunology.

[61] Chow A., Zhou W., Liu L., Fong M.Y., Champer J., van Haute D., Chin A.R., Ren X., Gugiu B.G., Meng Z. (2014). Macrophage immunomodulation by breast cancer-derived exosomes requires Toll-like receptor 2-mediated activation of NF-κB. Sci. Rep..

[62] Schulz E., Karagianni A., Koch M., Fuhrmann G. (2020). Hot EVs—How temperature affects extracellular vesicles. Eur. J. Pharm. Biopharm..

[63] Workman P., Aboagye E.O., Balkwill F., Balmain A., Bruder G., Chaplin D.J., Double J.A., Everitt J., Farningham D.A.H., Glennie M.J. (2010). Guidelines for the welfare and use of animals in cancer research. Br. J. Cancer.

[64] Reich M., Liefeld T., Gould J., Lerner J., Tamayo P., Mesirov J.P. (2006). GenePattern 2.0. Nat. Genet..

[65] Moscheni C., Malucelli E., Castiglioni S., Procopio A., de Palma C., Sorrentino A., Sartori P., Locatelli L., Pereiro E., Maier J.A. (2019). 3D Quantitative and Ultrastructural Analysis of Mitochondria in a Model of Doxorubicin Sensitive and Resistant Human Colon Carcinoma Cells. Cancers.

[66] Baxter E.W., Graham A.E., Re N.A., Carr I.M., Robinson J.I., Mackie S.L., Morgan A.W. (2020). Standardized protocols for differentiation of THP-1 cells to macrophages with distinct M(IFNγ+LPS), M(IL-4) and M(IL-10) phenotypes. J. Immunol. Methods.

