# Supplementary material for: The Educational Program of Macrophages toward a Hyperprogressive Disease-Related Phenotype Is Orchestrated by Tumor-Derived Extracellular Vesicles
Source: Int J Mol Sci. 2022 Dec 13;23(24):15802. doi: 10.3390/ijms232415802 (PMC9779478; doi:10.3390/ijms232415802)
Supplement: Supplementary file 1 [file ijms-23-15802-s001.zip › Supplementary Legends.pdf]

Table S1. Mutational landscape of NSCLC cell lines. Most relevant genetic alterations harbored by the five NSCLC cell lines utilized to polarize macrophages. mut: mutated, wt: wild-type, del: deletion.

Table S2. **List of differentially expressed transcripts between Group 1 and Group 2 BMDMs**. List of differentially expressed transcripts (DEGs) emerged by comparing Group 1 and Group 2 macrophage normalized gene profile data. Genes with FDR < 0.01 and FC ≤ -2 and ≥ 2 were considered DEGs.

Table S3. Lists of differentially expressed annotated transcripts up- or down-modulated in Group 1 macrophages compared to all the other experimental groups. Lists of DEGs (FDR < 0.01 and FC ≤ -2 and ≥ 2) resulted significantly up- or -down-regulated in Group 1 macrophages compared to every other experimental group (Group 2, M0, M1 and M2 macrophages).

Table S4. "Common UP in Group 1" and "Common DOWN in Group 1" gene lists. Lists of the 381 and 237 unique annotated transcripts resulted commonly up- and down-modulated in Group 1 BMDMs, respectively, in comparison to Group 2, M0, M1 and M2 macrophages.

Table S5. Size distribution analysis of H460-EVs. Size distribution of EVs isolated from H460 lung cancer cell line (NanoSight NS300 instrument - Malvern Panalytical). Mean ± SEM data derived from 7 replicates are shown.

Table S6. List of TaqMan® gene expression assays utilized for Real-time PCR analysis.

Figure S1. mRNA expression level of cytokines and chemokines in BMDMs exposed to NSCLC cell lines conditioned medium. (**A**) Hierarchical clustering based on the expression level of 15 cytokines and chemokines, retrieved from the "Common UP in Group 1" gene list, of BMDMs exposed to the different CMs and of M0, M1 and M2 macrophages. (**B**) Plot showing Il6 expression level on the x-axis and Il10 expression level on the y-axis. Data are presented as $2^{-\Delta Ct}$ as determined by Real-time PCR performed on the same samples utilized for microarray analysis.

Figure S2. Effect of protein and lipid removal from H460 CM on *Nupr1* and *Trib3* expression. mRNA level of *Nupr1* and *Trib3* genes, as determined by Real-Time PCR, after BMDMs exposure to H460 CMs in which proteins (heated-CMs) or lipids (lipid removed-CMs) were removed. Data were normalized to the housekeeping gene expression level (*β2m*) and analyzed by the comparative $2^{-\Delta\Delta Ct}$ method. Data are presented as the mean ± SEM. *p<0.05, **p<0.01, by unpaired t test or Mann–Whitney U test.

Figure S3. **Effect of H460-, PC9- and H1299-derived EVs on RAW264.7 mouse macrophage cell line**. mRNA level of *Il1β*, *Il6*, *Cd69* and *Lcn2* genes, as determined by Real-Time PCR, after RAW264.7 exposure to H460-, PC9- and H1299-derived EVs. Data were normalized to the housekeeping gene expression level (*β2m*) and analyzed by the comparative $2^{-\Delta Ct}$ method. Data are presented as the mean ± SEM. *p<0.05 by Mann–Whitney U test.

Figure S4. **Effect of H460-, PC9- and H1299-derived EVs on THP-1-derived human macrophages**. mRNA level of *IL1β*, *IL6*, *MARCO*, and *CD69* genes, as determined by Real-Time PCR, after THP-1-derived human macrophages exposure to H460-, PC9- and H1299-derived EVs. Data were normalized to the housekeeping gene expression level (*B2M*) and analyzed by the comparative $2^{-\Delta Ct}$ method. Data are presented as the mean ± SEM. *p<0.05 by Mann–Whitney U test.
