# Supplementary material for: The Educational Program of Macrophages toward a Hyperprogressive Disease-Related Phenotype Is Orchestrated by Tumor-Derived Extracellular Vesicles
Source: Int J Mol Sci. 2022 Dec 13;23(24):15802. doi: 10.3390/ijms232415802 (PMC9779478; doi:10.3390/ijms232415802)
Supplement: Supplementary file 1 [file ijms-23-15802-s001.zip › Table S1.pdf]

**Cell line**	***KRAS***	***LKB1***	***EGFR***	***TP53***	**HPD model**	**Ref.**
**H460**	mut	mut	wt	wt	Yes	[1]
**PC9**	wt	wt	mut	mut	Yes	[1]
**H1299**	wt	wt	wt	del	No	Unpublished data
**A549**	mut	mut	wt	wt	No	[2,3]
**Calu-1**	mut	wt	wt	del	NE	N/A

mut: mutated, wt: wild-type, del: deletion. NE: not evaluated; N/A: not applicable

References

1. Lo Russo, G.; Moro, M.; Sommariva, M.; Cancila, V.; Boeri, M.; Centonze, G.; Ferro, S.; Ganzinelli, M.; Gasparini, P.; Huber, V.; et al. Antibody-Fc/FcR Interaction on Macrophages as a Mechanism for Hyperprogressive Disease in Non-small Cell Lung Cancer Subsequent to PD-1/PD-L1 Blockade. *Clin. Cancer Res.* **2019**, *25*, 989–999, doi:10.1158/1078-0432.CCR-18-1390.
2. Kumar, S.; Ghosh, S.; Sharma, G.; Wang, Z.; Kehry, M.R.; Marino, M.H.; Neben, T.Y.; Lu, S.; Luo, S.; Roberts, S.; et al. Preclinical characterization of dostarlimab, a therapeutic anti-PD-1 antibody with potent activity to enhance immune function in in vitro cellular assays and in vivo animal models. *mAbs* **2021**, *13*, doi:10.1080/19420862.2021.1954136.
3. Moon, E.K.; Ranganathan, R.; Eruslanov, E.; Kim, S.; Newick, K.; O'Brien, S.; Lo, A.; Liu, X.; Zhao, Y.; Albelda, S.M. Blockade of Programmed Death 1 Augments the Ability of Human T Cells Engineered to Target NY-ESO-1 to Control Tumor Growth after Adoptive Transfer. *Clin. Cancer Res.* **2016**, *22*, 436–447, doi:10.1158/1078-0432.CCR-15-1070.
