# Supplementary material for: The Educational Program of Macrophages toward a Hyperprogressive Disease-Related Phenotype Is Orchestrated by Tumor-Derived Extracellular Vesicles
Source: Int J Mol Sci. 2022 Dec 13;23(24):15802. doi: 10.3390/ijms232415802 (PMC9779478; doi:10.3390/ijms232415802)
Supplement: Supplementary file 1 [file ijms-23-15802-s001.zip › Table S5 .pdf]

**Mean size (nm)**	128 ± 7
**Mode size[1] (nm)**	98 ± 4
**D10[2] (nm)**	80 ± 4
**D50[2] (nm)**	115 ± 5
**D90[2] (nm)**	194 ± 15
**EVs < 160 nm (%)**	80 ± 3

[1]Mode is the value that appears most often in a set of data values

[2]D10, D50 and D90 values indicate percent undersize. For example: 50% particles are 115 nm or smaller.
