# Supplementary material for: The Educational Program of Macrophages toward a Hyperprogressive Disease-Related Phenotype Is Orchestrated by Tumor-Derived Extracellular Vesicles
Source: Int J Mol Sci. 2022 Dec 13;23(24):15802. doi: 10.3390/ijms232415802 (PMC9779478; doi:10.3390/ijms232415802)
Supplement: Supplementary file 1 [file ijms-23-15802-s001.zip › Table S6.pdf]

Mouse TaqMan® gene expression assays	
**Gene Symbol**	**Assay ID**
Il1β	Mm00434228_m1
Il6	Mm00446190_m1
Marco	Mm00440265_m1
Cd69	Mm01183378_m1
Lcn2	Mm01324470_m1
Il10	Mm01288386_m1
Cxcr4	Mm01996749_s1
Pparg	Mm00440940_m1
Timp2	Mm00441825_m1
Actin1	Mm01304398_m1
Bgn	Mm01191753_m1
Nupr1	Mm00498104_m1
Trib3	Mm00454879_m1
β2m	Mm00437762_m1

Human TaqMan® gene expression assays	
**Gene Symbol**	**Assay ID**
IL1β	Hs01555410_m1
IL6	Hs00174131_m1
MARCO	Hs00198937_m1
CD69	Hs00934033_m1
LCN2	Hs01008571_m1
β2M	Hs00187842_m1
