# Supplementary figures and images for: The Educational Program of Macrophages toward a Hyperprogressive Disease-Related Phenotype Is Orchestrated by Tumor-Derived Extracellular Vesicles
Source: Int J Mol Sci. 2022 Dec 13;23(24):15802. doi: 10.3390/ijms232415802 (PMC9779478; doi:10.3390/ijms232415802)

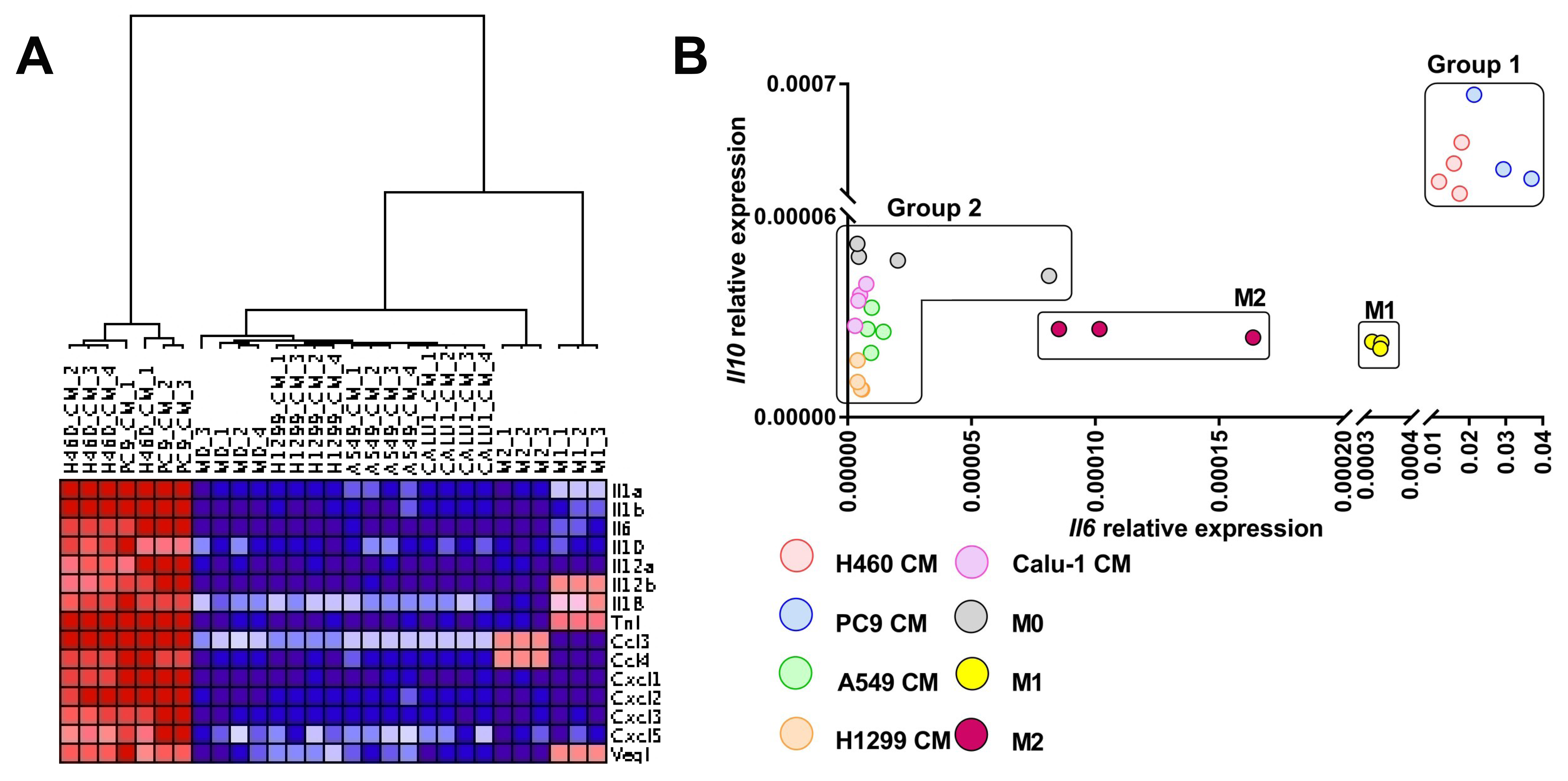

Supplement: Supplementary file 1 [file ijms-23-15802-s001.zip › Figure S1.tif]

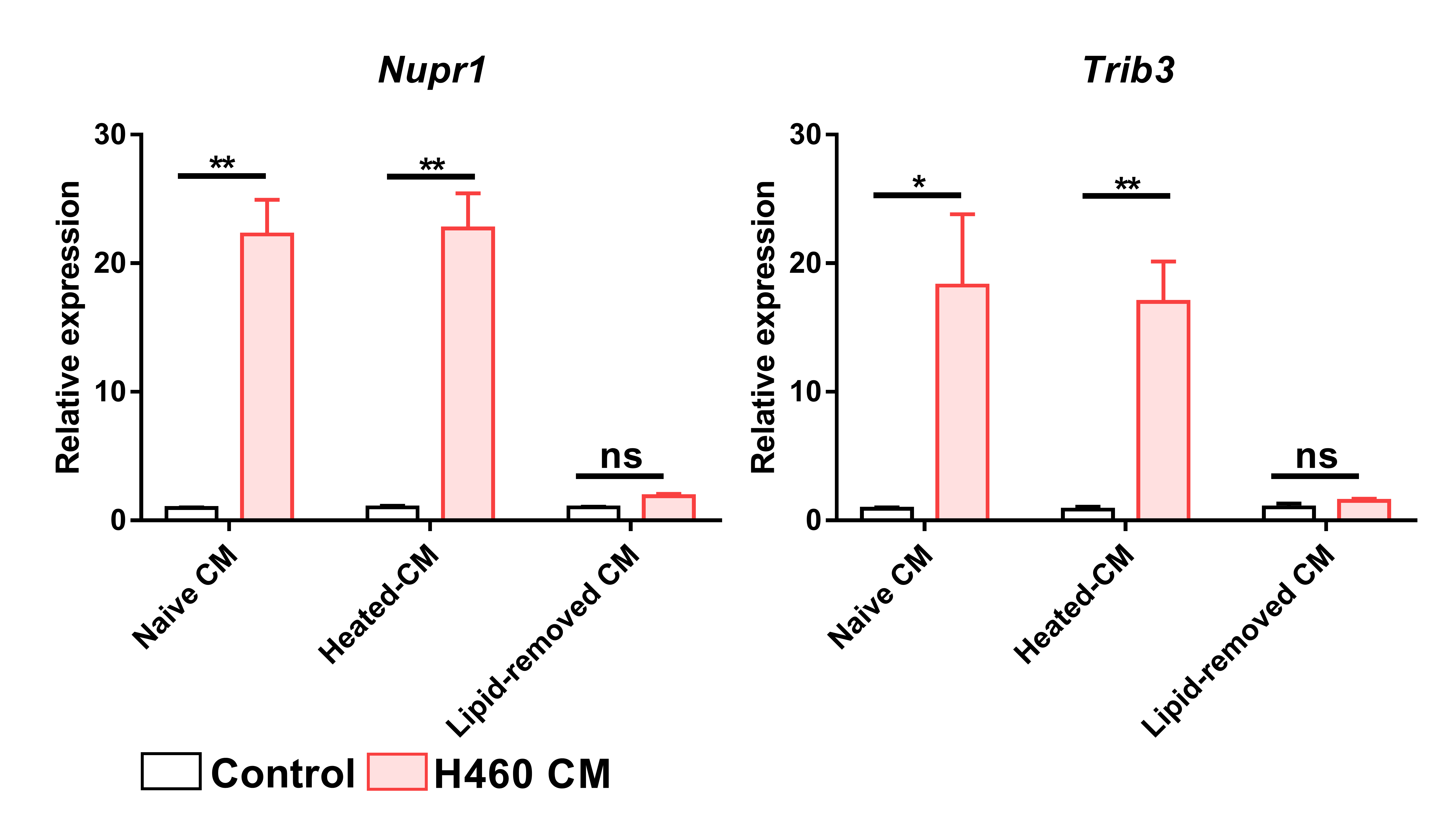

Supplement: Supplementary file 1 [file ijms-23-15802-s001.zip › Figure S2.tif]
